# Differential Proteomic Analysis of Astrocytes and Astrocytes-Derived Extracellular Vesicles from Control and Rai Knockout Mice: Insights into the Mechanisms of Neuroprotection

**DOI:** 10.3390/ijms22157933

**Published:** 2021-07-25

**Authors:** Tommaso Montecchi, Enxhi Shaba, Domiziana De Tommaso, Fabrizio Di Giuseppe, Stefania Angelucci, Luca Bini, Claudia Landi, Cosima Tatiana Baldari, Cristina Ulivieri

**Affiliations:** 1 Department of Life Sciences, University of Siena, Via Aldo Moro, 2, 53100 Siena, Italy; tommaso.montecchi@gmail.com (T.M.); detommasodomiziana@gmail.com (D.D.T.); 2 Functional Proteomics Laboratory, Department of Life Sciences, University of Siena, Via Aldo Moro, 2, 53100 Siena, Italy; shaba3@student.unisi.it (E.S.); luca.bini@unisi.it (L.B.); 3 Centre of Advanced Studies and Technoloy, Dentistry and Biotechnology and Proteomics Unit, Department Medical, Oral & Biotechnological Sciences, “G. d’Annunzio”, University of Chieti-Pescara, Via dei Vestini, 31, 66100 Chieti, Italy; f.digiuseppe@unich.it (F.D.G.); s.angelucci@unich.it (S.A.)

**Keywords:** astrocytes, molecular adaptor, IL-17, proteomics, extracellular vesicles, HIF-1α

## Abstract

Reactive astrocytes are a hallmark of neurodegenerative disease including multiple sclerosis. It is widely accepted that astrocytes may adopt alternative phenotypes depending on a combination of environmental cues and intrinsic features in a highly plastic and heterogeneous manner. However, we still lack a full understanding of signals and associated signaling pathways driving astrocyte reaction and of the mechanisms by which they drive disease. We have previously shown in the experimental autoimmune encephalomyelitis mouse model that deficiency of the molecular adaptor Rai reduces disease severity and demyelination. Moreover, using primary mouse astrocytes, we showed that Rai contributes to the generation of a pro-inflammatory central nervous system (CNS) microenvironment through the production of nitric oxide and IL-6 and by impairing CD39 activity in response to soluble factors released by encephalitogenic T cells. Here, we investigated the impact of Rai expression on astrocyte function both under basal conditions and in response to IL-17 treatment using a proteomic approach. We found that astrocytes and astrocyte-derived extracellular vesicles contain a set of proteins, to which Rai contributes, that are involved in the regulation of oligodendrocyte differentiation and myelination, nitrogen metabolism, and oxidative stress. The HIF-1α pathway and cellular energetic metabolism were the most statistically relevant molecular pathways and were related to ENOA and HSP70 dysregulation.

## 1. Introduction

Astrocytes are the predominant cell type in the central nervous system (CNS) which control the homeostasis of the CNS through the regulation of extracellular ion concentration and the secretion of a wide array of soluble factors including neurotrophic factors and neurotransmitters [1]. In addition, similar to other cell types, astrocytes release extracellular vesicles (EVs) under both physiological and pathological conditions which further contribute to shape the CNS microenvironment [2]. As components of the glia limitants and by expressing specialized channel-forming proteins (AQP4, Kir4.1 and Connexin43), astrocytes also regulate the diffusion of soluble factors across the blood brain barrier (BBB) [3]. The recent demonstration that EVs are able to cross the BBB and that astrocytes are the main secretory cells of the CNS [4] further supports the notion that astrocytes are key determinants of brain function.

It is now widely accepted that astrocyte reaction to CNS insults is heterogeneous and depends on physiological context, type of injury, location in the CNS, and microenvironment. Indeed, when CNS injury or disease occurs, astrocytes react by changing morphological, biochemical, transcriptional, and functional programs as a result of a combination of environmental cues and intrinsic features [5]. The signals and associated signaling pathways that drive astrocyte reaction are at present largely unknown, but a mechanism by which activated microglia through the secretion of IL-1α, TNF, and C1q induces neurotoxic astrocytes has been described [6].

In recent years astrocytes have emerged as crucial modulators of inflammation during multiple sclerosis (MS), a CNS-affecting autoimmune disease, and experimental autoimmune encephalomyelitis (EAE), the mouse model of human MS [7,8,9]. Relevant to MS, depletion of astrocytes either genetically or following ganciclovir treatment in the EAE animal model resulted in different outcomes depending on the phase of disease. Inhibition of astrocyte reaction worsens the EAE course during the early phase of disease, while in the chronic phase it improves the clinical signs, underscoring the complex and multifaceted role played by these cells in MS [9]. This view of astrocytes as cells able to protect or promote disease depending on the environmental context and disease stage is challenging and supports the rationale to consider astrocytes as targets for the development of therapies for neurodegenerative disease [10]. This has been hampered to date by the lack of information on the molecular mechanisms driving astrocyte reactivity and the fact that astrocytes have been only recently identified as central modulators of neurodegeneration.

At present, the “omics” approaches including proteomics, spatial transcriptomics, and single-cell RNA sequencing represent the gold standard to understand the impact of external stimuli and cell-intrinsic characteristics on astrocyte responses in a given context and to identify specific molecular markers [11,12]. In this context, a seminal report by Wheeler and colleagues identified several subpopulations of astrocytes in EAE mice that differed from those found in healthy mice by single-cell transcriptomic analysis [13]. In particular, a subpopulation of astrocytes characterized by the activation of the pro-inflammatory and neurotoxic pathways such as the unfolding protein response, the activation of NF-κB, and the inducible nitric oxide synthase (iNOS) pathways has been identified as the pathogenic and most expanded subpopulation during EAE [13].

We demonstrated that Rai, a member of the Shc family of protein adaptors that negatively regulates T cell activation and Th17 cell development [14,15], is expressed in astrocytes where it promotes the production of pro-inflammatory mediators (IL-6, NO) induced by Th17 cells. Interestingly, EAE mice lacking Rai showed amelioration of disease severity characterized by delayed onset, lower clinical score, and reduced demyelination compared with control EAE mice. The reduced degree of demyelination was associated with a reduced activation of astrocytes, supporting the notion that Rai expression in astrocytes is an important determinant in MS immunopathology [16]. We recently demonstrated that the ability of astrocytes lacking Rai to control the pathogenic potential of autoreactive T cells involves the conversion of extracellular ATP to the immunosuppressive molecule adenosine through the enhancement of CD39 ectonucleotidase activity [17]. Moreover, we showed that astrocytes lacking Rai skew towards a neuroprotective phenotype in response to encephalitogenic T cells, both in vitro and in EAE mice [17]. Together, these findings underscore the multifaceted role played by Rai in astrocyte reactivity during EAE, suggesting the possibility that targeting Rai expression in astrocytes may represent a strategy to impact on the course of disease.

Here, we investigated whether Rai has an impact on the astrocyte proteome both in basal conditions and following activation with the pro-inflammatory cytokine IL-17. Additionally, since astrocyte-derived extracellular vesicles (ADEV) play a crucial role in shaping the surrounding microenvironment both in physiological and pathological conditions [2], we investigated whether Rai impinges on the proteome of ADEVs under the same conditions.

## 2. Results

### 2.1. Rai Participates in the Astrocyte Response to IL-17 and IFNγ through the Activation of NF-κB

**Figure 1:**
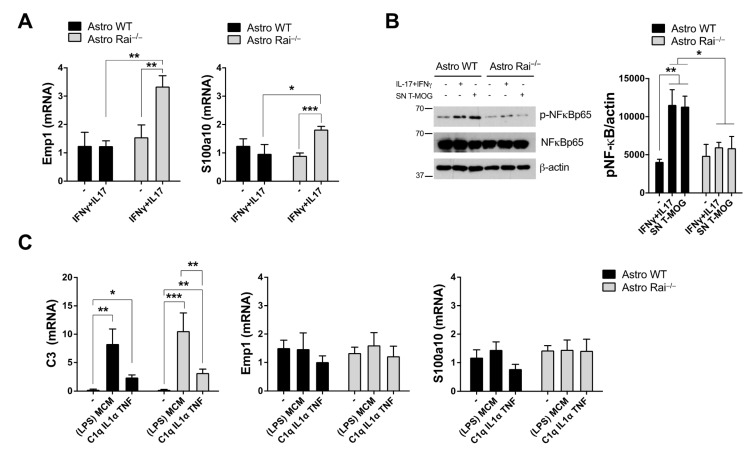
Rai signaling controls astrocyte reaction to soluble factors secreted by MOG-specific T cells, but not by microglia. (**A**) qRT-PCR analysis of Emp1 and S100a10 transcripts in WT (Astro WT) and Rai^−/−^ (Astro Rai^−/−^) astrocytes untreated (-) or treated for 24 h with a combination of IFNγ (10 ng/mL) and IL-17 (50 ng/mL) (IFNγ + IL-17). Data are presented as mean value ± SD (*n* = 4); (**B**) Immunoblot analysis of phosphorylated NF-κB in lysates of WT (Astro WT) and Rai^−/−^ (Astro Rai^−/−^), astrocytes untreated or treated for 15 min at 37 °C with culture supernatants from MOG-specific T cells or as in (**A**). Actin and NF-κB were used as loading control. The histogram shows the quantification by densitometric analysis of the levels of phosphorylated NF-κB relative to actin (*n* = 4); (**C**) qRT-PCR analysis of C3, Emp1, and S100a10 transcripts in WT (Astro WT) and Rai^−/−^ (Astro Rai^−/−^), astrocytes untreated or treated for 24 h at 37 °C with culture supernatants from LPS-activated microglia (LPS-MCM) or with a combination of IL-1α (3 ng/mL), TNF (30 ng/mL), and C1q (400 ng/mL). Data are presented as mean value ± SD (*n* = 5 for LPS-MCM, *n* = 3 for IL-1α, TNF, and C1q). *** *p* < 0.001, ** *p* < 0.01, * *p* < 0.05.

We recently provided evidence that conditioned media from MOG-specific T cells induced the acquisition of a neuroprotective phenotype by astrocytes lacking the molecular adaptor Rai [17]. To determine whether the Th17 signature cytokines, IL-17 and IFNγ are responsible for this effect, control and Rai^−/−^ astrocytes were treated with these recombinant cytokines and the expression levels of transcripts known to be upregulated in neuroprotective astrocytes were measured by qRT-PCR. Rai^−/−^ astrocytes showed a significant upregulation of the neuroprotective-specific transcripts Emp1 and S100a10 compared with control astrocytes (Figure 1A), consistent with the results obtained following treatment with conditioned media from MOG-specific T cells [17]. Analysis of the activation status of NF-kB, a key transcription factor in cytokine signaling and a known driver of the proinflammatory response of astrocytes [18], showed that it was completely inhibited in Rai^−/−^ astrocytes both following stimulation with IL-17 and IFNγ, and in the presence of conditioned media from encephalitogenic T cells (Figure 1B) suggesting that Rai drives the astrocyte response to inflammatory soluble factors secreted by T cells through the activation of NF-kB. Of note, treatment of control and Rai^−/−^ astrocytes with known inducers of astrocyte neurotoxicity, namely conditioned medium from LPS-activated microglia or a combination of the microglia-derived soluble factors, IL-1α, TNF, and C1q [6], resulted in a strong upregulation of the neurotoxic-specific transcripts C3 not only in control astrocytes as previously reported [6] but also in Rai-deficient astrocytes (Figure 1C). At variance, Rai deletion did not affect the levels of Emp1 and S100a10 mRNA under these conditions (Figure 1C) indicating that Rai is a key participant in the signaling pathway controlling the astrocyte response to T cells, but not to microglia.

### 2.2. Differential Proteome Profile of Control and Rai^−/−^ Astrocytes

While our data suggest that the T cell-dependent detrimental astrocyte reaction is supported by Rai (Figure 1 and [16]), whether astrocytes lacking Rai gain some protective functions that contribute to cell-autonomous neuroprotection is unknown. Thus, we investigated the impact of Rai on the homeostatic functions of astrocytes and on the functional changes in response to IL-17, the signature cytokine of Th17 cells responsible for astrogliosis during EAE [19] whose signaling is impaired by Rai deficiency [16], by large scale comparison of the proteome profiles of control and Rai^−/−^ astrocytes.

**Figure 2:**
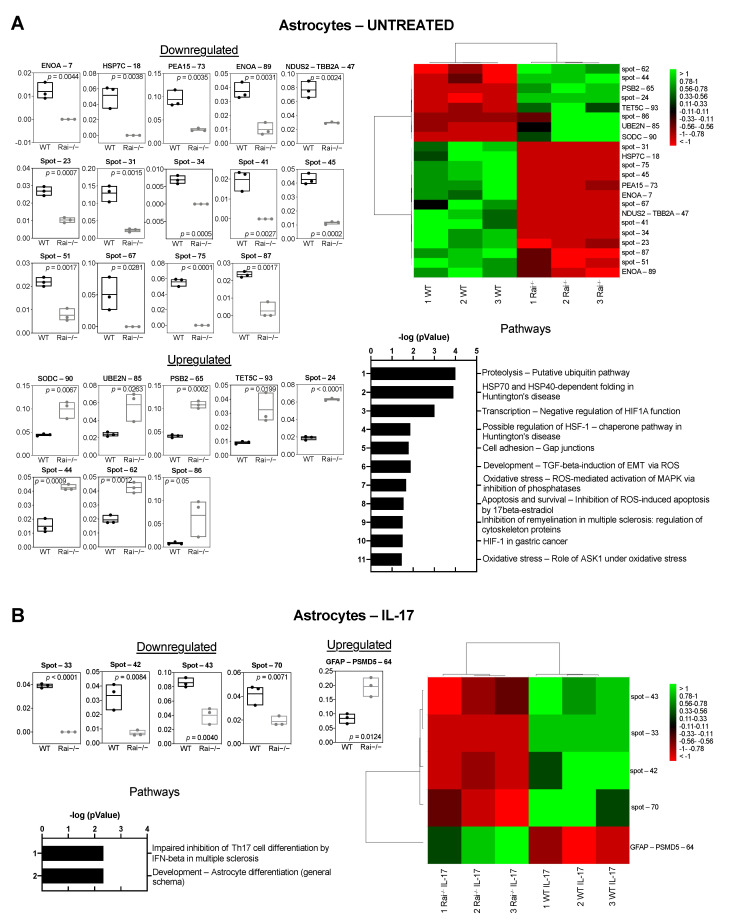
Differential proteomic analysis of control and Rai^−/−^ astrocytes. Supervised hierarchical clustering heat map (**left**) and floating bars (min to max) (**right**) of differentially abundant proteins found in untreated (**A**) and IL-17-treated (**B**) astrocytes purified from control (WT) and Rai^−/−^ mice (*n* = 3 WT, 3 Rai^−/−^). Heat maps: columns correspond to individual preparations of astrocytes and row to spot identity (protein name, or spot number for not identified spots) is indicated on the right. Color scale (from high value in green to low value in red) illustrates % volume values of the statistically significant differentially abundant spots. The most relevant pathway maps reported by MetaCore, based on all differentially expressed proteins and prioritized according to their statistical significance (*p* ≤ 0.001), are shown for each condition.

We first determined whether Rai deficiency impacts the homeostatic proteome of astrocytes. Under basal conditions, 2D-Electrophoresis (2DE) and image analysis revealed a mean of 2914 spots per gel. The Kruskall–Wallis test for multiple comparisons and false discovery rate control identified 22 differentially abundant spots in astrocytes of control and Rai^−/−^ mice. All 22 electrophoretic spots were subjected to protein identification by mass spectrometry, only 9 of which were identified. Differential spots are shown in Figure S1A. Principal component analysis performed to evaluate variations in the datasets showed that control samples were well distinct from Rai^−/−^ samples (Figure S2A). Among the differential spots that were up-regulated in Rai^−/−^ astrocytes we found proteasome subunit beta type 2 (PSB2), ubiquitin-conjugating enzyme E2 N (UBE2N), superoxide dismutase (SODC), and terminal nucleotidyl transferase 5C (TET5C), while among the down-regulated spots we found alpha enolase (ENOA), heat shock cognate 71 kDa protein (HSP7C), NADH dehydrogenase iron-sulfur protein 2 mitochondrial (NDUS2), tubulin beta-2A chain (TBB2A), and astrocytic phosphoprotein PEA-15 (PEA15) (Figure 2A box plot, Table S1A). Hierarchical clustering analysis of proteins significantly altered in abundance demonstrated consistent replicate samples within one group and difference between control and Rai^−/−^ astrocytes (Figure 2A heat map).

In order to investigate the global changes in protein composition induced by IL-17 and the impact of Rai expression on these changes, we next compared the proteome of IL-17-treated control and Rai^−/−^ astrocytes. A mean of 2252 spots was detected in each gel (Figure S1B). Through image and statistical analyses, we identified glial fibrillary acidic protein (GFAP) among the proteins that were up-regulated in Rai^−/−^ astrocytes treated with IL-17 compared to their wild-type counterparts (Figure 2B, Table S1A).

Enrichment analysis performed using the MetaCore 6.8 network building tool (Clarivate Analytics, Philadelphia, PA, USA) shed light on the molecular pathways associated to the identified differential proteins (Figure 2). In particular, differential proteins found under basal conditions were involved in some potentially relevant pathways such as the putative ubiquitin pathway, the HSP70-dependent folding pathway, the HSP70/TLR signaling pathway, the negative regulation of HIF-1α function, and TGF-beta-induction of EMT via ROS (Figure 2A, Table S2). GFAP, which is involved in astrocyte differentiation pathway, was the only differentially identified protein in IL-17-treated samples.

### 2.3. Differential Proteome Profile of Control and Rai^−/−^ Astrocytes-Derived Extracellular Vesicles

Our finding that astrocytic Rai is required for the generation of a pro-inflammatory CNS microenvironment [16,17], together with the key role played by ADEVs in shaping the surrounding microenvironment [2], prompted us to explore the impact of Rai on the ADEV proteome both under basal condition and in response to IL-17.

**Figure 3:**
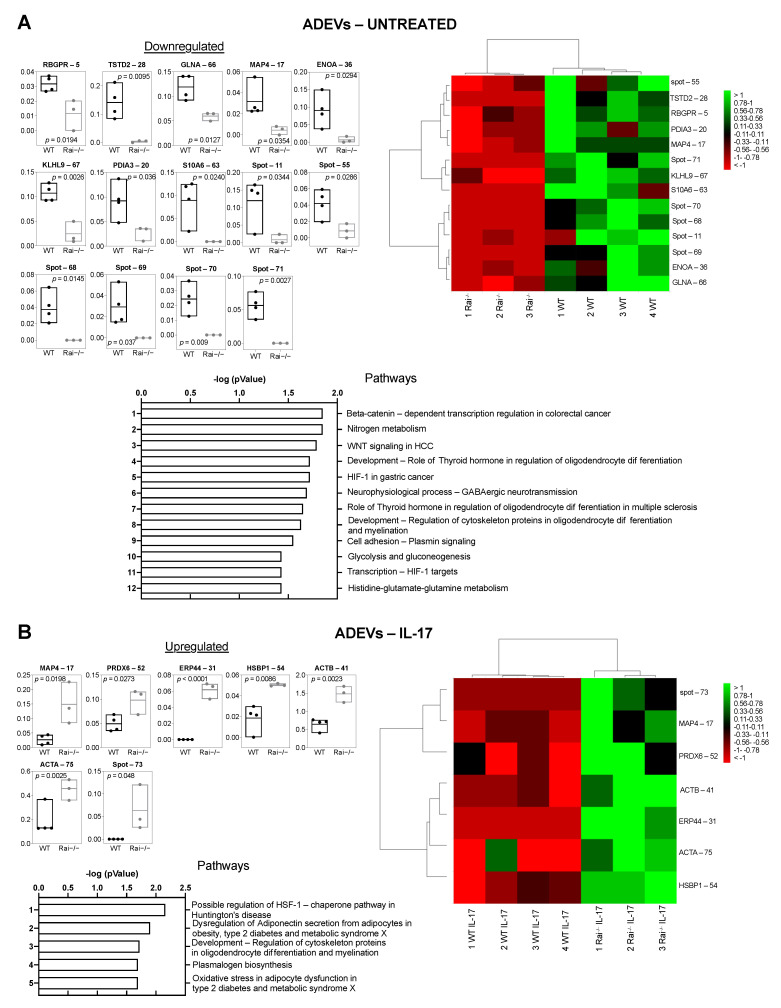
Differential proteomic analysis of control and Rai^−/−^ ADEVs. Supervised hierarchical clustering heat map (left) and floating bars (min to max) (right) of differentially abundant proteins found in ADEVs purified from the cell media of control (WT) and Rai^−/−^ astrocytes untreated (**A**) and treated with IL-17 (**B**) (*n* = 4 WT, 3 Rai^−/−^). Heat maps: columns correspond to individual preparations of ADEVs and row to spot identity (protein name, or spot number for not identified spots). Color scale (from high value in green to low value in red) illustrates % volume values of the statistically significant differentially abundant spots. The most relevant pathway maps reported by MetaCore, based on all differentially expressed proteins and prioritized according to their statistical significance (*p* ≤ 0.001), are shown for each condition.

ADEVs from both untreated and IL-17-treated control and Rai^−/−^ astrocytes were isolated using a sequential ultracentrifugation and the protein content was characterized by 2DE and image analysis. Under basal conditions, we found a mean of 694 spots per gel. The quantitative comparison of the normalized % volume values for each spot revealed 14 differentially abundant spots validated by statistics in Rai^−/−^ ADEVs compared with WT ADEVs. Differential spots are shown in Figure S3A, eight of which were identified by mass spectrometry (Figure 3A box plot, Table S1B). Principal component analysis revealed that WT and Rai^−/−^ ADEVs were well separated (Figure S2A). Identified proteins that were down-regulated in Rai^−/−^ ADEVs compared with WT ADEVs were Rab3 GTPase-activating protein non-catalytic subunit (RBGPR), protein disulfide-isomerase A3 (PDIA3), thiosulfate sulfurtransferase/rhodanese-like domain-containing protein 2 (TSTD2), alpha-enolase (ENOA), glutamine synthetase (GLNA), kelch-like protein 9 (KLHL9), and microtubule-associated protein 4 (MAP4), while S10A6 was present only in WT ADEVs (Figure 3A box plot, Table S1B).

A mean of 660 spots per gel was found in control and Rai^−/−^ ADEVs following IL-17 treatment. Seven differentially abundant spots were extrapolated and are shown in supplementary Figure 3B. Six were identified (Figure 3B, Table S1B). These include actin cytoplasmic 1 (ACTB), peroxiredoxin-6 (PRDX6), heat shock factor-binding protein 1 (HSBP1), actin aortic smooth muscle (ACTA), and MAP4, which were enriched in IL-17-Rai^−/−^ ADEVs compared with IL-17-WT ADEVs. Moreover, the endoplasmic reticulum resident protein 44 (ERP44) was detected only in IL-17-Rai^−/−^ ADEVs (Figure 3B box plot). MAP4 is the only protein shared in these conditions (basal versus IL-17). Interestingly, it was found to be enriched in IL-17 Rai^−/−^ ADEVs compared with IL-17-WT ADEVs and conversely, under basal conditions, it was less abundant in Rai^−/−^ ADEVs compared with WT ADEVs (Figure 3 box plot). Principal component analysis and hierarchical clustering of significant differential spots showed that WT ADEVs and Rai^−/−^ ADEVs cluster furthest away from each other (Figure 3B heat map, Figure S2B). Moreover, in basal conditions, we observed a predominance in low-abundance proteins in Rai^−/−^ ADEVs with respect to WT ADEVs, while in IL-17 stimulated condition, we observed an opposite behavior, indicating that Rai impinges on the ADEV proteome in both conditions (Figure 3).

Pathway analyses of the identified proteins under basal conditions highlighted their involvement in several pathways, including beta-catenin dependent transcription regulation, nitrogen metabolism, Wnt signaling of oligodendrocyte differentiation, GABAergic neurotransmission, HIF-1-dependent transcription, glycolysis and gluconeogenesis, and histidine-glutamate-glutamine metabolism, and plasmin signaling.

On the other hand, pathway analysis of the IL-17-treated condition showed their involvement in the regulation of the HSF-1/chaperone pathway, dysregulation of adiponectin secretion, regulation of cytoskeleton proteins in oligodendrocytes differentiation, plasmalogen biosynthesis, and oxidative stress (Figure 3, Table S3).

**Figure 4:**
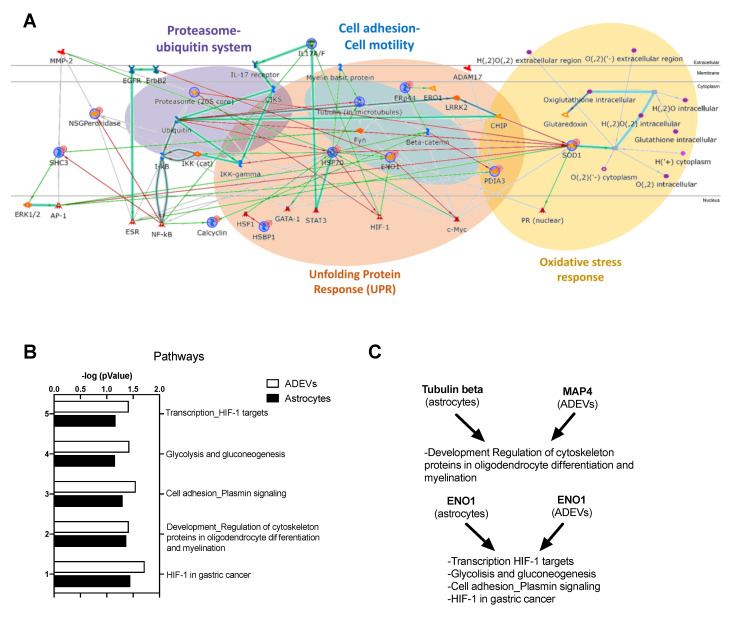
Protein network and pathways influenced by Rai in astrocytes and ADEVs. (**A**) Protein network analysis by using all the proteomic data obtained in WT astrocytes and ADEVs and those obtained in Rai^−/−^ astrocytes and Rai^−/−^ ADEVs. Proteasome (20 S core), HSP70, ENOA, and SOD1 are central functional hubs; (**B**) Pathway analysis comparison of the differential proteins found in astrocytes and ADEVs; (**C**) Association of differential proteins to the molecular pathways resulting from pathway analysis comparison of astrocytes and ADEVs.

Since the ADEV proteome might reflect the cellular changes induced by Rai, we performed a protein network using all the proteomic data obtained in wild type astrocytes and ADEVs and those obtained in Rai^−/−^ astrocytes and Rai^−/−^ ADEVs to identify the most relevant molecular pathways influenced by Rai. The protein network, presented in Figure 4A, showed the proteasome (20S core), HSP70, ENO1, and SOD1 as central functional hubs. These proteins are relevant players in the proteasome ubiquitin system, unfolding protein response, ECM remodeling/cell adhesions, and oxidative stress response, respectively.

In addition, a pathway analysis comparison of differential proteins between astrocytes and ADEVs was performed. HIF-1α pathway and cellular energetic metabolism were among the most statistically significant shared relevant molecular pathways, together with the involvement of regulation of cytoskeleton proteins in oligodendrocyte differentiation and myelination (Figure 4B, Table S4). In particular, ENO1 dysregulation was related to HIF-1 pathway, glycolysis and gluconeogenesis, and plasmin signaling, while tubulin beta and MAP4 influenced the regulation of cytoskeleton proteins in oligodendrocytes differentiation and myelination (Figure 4C, Table S4).

### 2.4. ENOA and HSP70 Provide New Insights into the Response of Rai^−/−^ Astrocytes to IL-17

**Figure 5:**
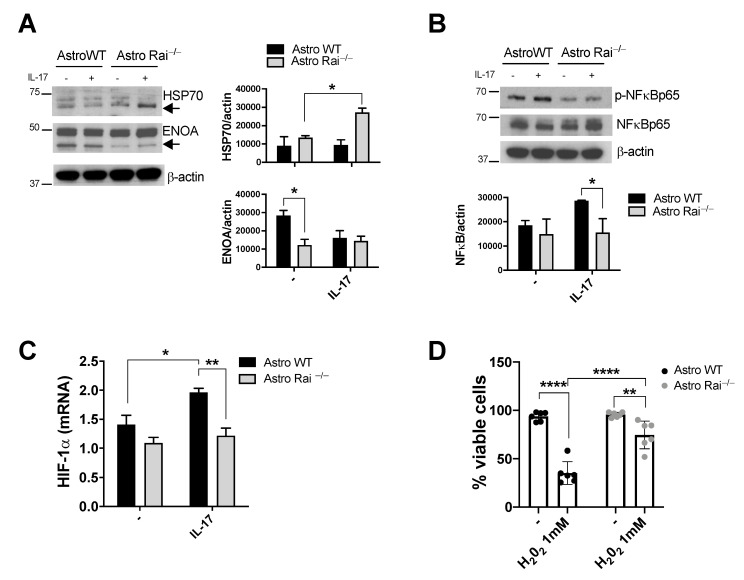
Rai supports the IL-17-dependent neuroinflammatory response in astrocytes through the activation of NFκB-HIF-1α pathway and the inhibition of HSP70 upregulation. (**A**) Immunoblot analysis of HSP70 and ENOA in lysates of WT (Astro WT) and Rai^−/−^ (Astro Rai^−/−^) astrocytes untreated or treated with IL-17 (50 ng/mL) for 24 h; (**B**) Immunoblot analysis of phosphorylated and total NF-κB in lysates of WT (Astro WT) and Rai^−/−^ (Astro Rai^−/−^) astrocytes treated with IL-17 for 15 min at 37 °C or left untreated. Histograms showed in (**A**) and (**B**) represent the quantification by densitometric analysis of the levels of the indicated proteins relative to actin (*n* = 3); (**C**) qRT-PCR analysis of HIF-1α transcripts in WT (Astro WT) and Rai^−/−^ (Astro Rai^−/−^,) astrocytes treated as in (**A**). Data are presented as mean value ± SD (*n* = 3); (**D**) Flow cytometric analysis of WT (Astro WT) and Rai^−/−^ (Astro Rai^−/−^,) astrocytes treated with 1 mM H_2_O_2_ for 24 h at 37 °C and stained with PI immediately before the acquisition. The graph shows the mean value ± SD of the percentage of PI negative cells (viable cells) (*n* = 6). **** *p* < 0.0001, ** *p* < 0.01, * *p* < 0.05.

The characterization of the proteomes of both astrocytes and ADEVs suggests that the differentially abundant proteins cooperate in specific molecular pathways, such as the HIF-1α transcriptional pathway, the unfolding protein response, cellular energetic metabolism, the proteasome-ubiquitin system, cell adhesion-cell motility, and oxidative stress. Among the differentially expressed proteins, two of these, namely ENOA and HSP7C, a member of the HSP70 family, attracted our attention because of the involvement of ENOA in the regulation of ECM degradation, ROS and NO production [20], and the ability of HSP70 to modulate cytokine-dependent NF-κB activation and NO production in astrocytes [21,22]. Control and Rai^−/−^ astrocytes were treated for 24 h with IL-17 or left untreated, and HSP70 and ENOA protein levels were analyzed by immunoblot. ENOA was found to be significantly reduced in astrocytes lacking Rai under basal conditions compared with control astrocytes (Figure 5A). In contrast, Rai deficiency resulted in a significant upregulation of HSP70 following IL-17 treatment compared with control astrocytes (Figure 5A). Of note, reduced NF-κB activation was observed in astrocytes lacking Rai compared with control astrocytes following IL-17 treatment (Figure 5B).

Pathway analysis comparison highlighted HIF1-α as a crucial transcription factor linked to both HSP70 and ENOA (Figure 4). HIF1-α, whose expression is controlled by NF-κB [23], promotes the expression of iNOS and ENOA in response to cytokine stimulation and hypoxia, respectively [24,25]. We therefore compared HIF-1α mRNA levels in Rai^−/−^ and control astrocytes. As opposed to WT astrocytes, Rai^−/−^ astrocytes did not upregulate HIF-1α mRNA levels following IL-17 treatment, consistent with the reduced activation of NF-κB (Figure 5C).

High levels of ROS, resulting at least in part from the dysregulated antioxidant function of astrocytes, are found in both EAE and MS lesions and are responsible for neurodegeneration [26,27]. Our proteomic results revealed that many of the proteins differentially expressed between control and Rai^−/−^ astrocytes are involved in the cellular oxidative stress response (Figure 4). To address whether Rai participates in the response of astrocytes to oxidative stress, control and Rai^−/−^ astrocytes were added with H_2_O_2_ and cultured for 24 h, followed by PI cell viability assay. H_2_O_2_ treatment caused a significant reduction in cell viability of control astrocytes at 1000 mM when compared with untreated samples. Rai deficiency prevented the decrease in cell viability of astrocytes upon H_2_O_2_-induced oxidative stress when compared with controls (Figure 5D), indicating that Rai deletion protects astrocytes from extracellular ROS.

## 3. Discussion

MS is an autoimmune neurodegenerative disease of the CNS characterized by the presence of demyelinated lesions in the brain and spinal cord and local inflammatory reaction [28]. How inflammation is connected to demyelination in MS is still an open question and several mechanisms have been proposed [29]. The low number of lymphocytes found at the site of active demyelination [29], as well as the finding that active demyelination can occur far from the immune cell infiltrates [30], led to speculation that soluble factors released by immune cells drive tissue injury through the direct or indirect activation of nearby cells, including microglia and astrocytes which are present in MS lesions [29,31]. Importantly, a direct link between immune cell-induced astrocyte response and neuronal and oligodendroglial cell death has been demonstrated in MS [18]; however, we still lack a full understanding of how astrocyte dysregulation drives disease. Although the acquisition of a neurotoxic state by astrocytes has been documented in response to soluble factors released by LPS-activated microglia [6], little is known about the nature of signals coming from infiltrating T cells during MS and their influence on astrocyte function. We recently identified Rai as a key signaling molecule in the control of the astrocyte reaction to conditioned media from encephalitogenic T cells [17] and here we further corroborate a role for Rai in the astrocyte response to the Th17 cell-secreted cytokines IL-17 and IFNγ, but not to pro-inflammatory mediators secreted by activated microglia. Importantly, here, we found an association between Rai and the activation of the transcription factor NF-κB in the control of the astrocyte reaction to soluble factors released by encephalitogenic T cells.

Proteomic analysis performed on astrocytes indicated a specific contribution of Rai in neurotoxic pathways such as the unfolded protein response, which has been previously identified, together with the activation of NF-κB, as one of the main pathways implicated in the pathogenic activities of astrocyte in EAE and MS [13]. Moreover, our analysis identified the proteasome-ubiquitin system, which is the main pathway for protein degradation in mammals, as a pathway influenced by Rai (Figure 4). Heat shock proteins are molecular chaperones responsible for the proper folding of proteins under physiological condition and in preventing protein aggregation and cell death following cellular stress, including hypoxia, oxidative stress, and exposure to inflammatory cytokines [32]. Among HSPs, HSP70 over-expression in astrocytes has been shown to promote neuroprotection by directly interacting with and inhibiting NF-κB, which in turn suppresses inducible NO synthase and pro-inflammatory cytokine expression [21]. Interestingly, upregulation of HSP70 in MS lesions has been suggested as a mechanism to counteract the inflammatory and oxidative microenvironment that is characteristic of the disease [32,33,34]. Our finding that HSP70 is upregulated in Rai^−/−^ astrocytes following IL-17 treatment paralleled by NF-κB inhibition highlights a role for Rai in the modulation of the IL-17-dependent unfolded protein response and proteasome-ubiquitin system in astrocytes. Moreover, the enhanced ability of Rai^−/−^ astrocytes to survive oxidative stress compared with control astrocytes further highlights the contribution of astrocytic Rai to neurodegeneration, at least in part by restraining the upregulation of HSP70.

Hypoxia-inducible factors-1α (HIF-1α) is a master transcription factor controlling cellular responses to hypoxia [35,36]. Beside hypoxia, HIF-1α upregulation can be induced by ROS, RNS, and pro-inflammatory cytokines, the latter through the activation of NF-κB [23,35]. In astrocytes, IL-17-dependent upregulation of HIF-1α has been demonstrated to contribute to the production of the pro-inflammatory cytokines IL-1β and IL-6 [36]. Moreover, HIF-1α-dependent upregulation of iNOS expression following TNF, IL-1, and IFNγ treatment has been recently shown in primary human astrocytes under normoxic condition [24]. We have previously shown that Rai deficiency in astrocytes impairs the production of IL-6 and iNOS following IL-17 and IFNγ treatment, respectively [16]. Here we show that the IL-17-dependent expression of HIF-1α is inhibited in Rai^−/−^ astrocytes. These data, together with the finding that Rai modulates NF-κB activation and HIF-1α expression in T cells under hypoxia [37], suggest that the mechanism by which IL-17 promotes the pro-inflammatory function of astrocytes depends at least in part on the activation of the Rai/NF-κB/HIF-1α axis. Of note, during neuroinflammation hypoxia has been shown to increase oxidative stress and to contribute to brain barrier dysfunction [38]. In addition, our pathway analysis results indicate that ENOA is correlated to HIF-1α activity. This multifunctional glycolytic enzyme migrates from the cytoplasm to the cell surface of macrophages, microglia, and astrocytes under inflammatory conditions to promote ECM degradation and the production of pro-inflammatory mediators including cytokines (IL-6, IL-1β, TNF-β, and TNF-α), chemokines (MCP-1 and MIP-1α), reactive oxygen species (ROS), and nitric oxide (NO) [20]. Accordingly, we found that ENOA expression is impaired in Rai^−/−^ astrocytes (Figure 5A), further supporting the notion that Rai-driven HIF-1α activation may be responsible for astrocyte reprogramming towards a neurotoxic phenotype.

CNS inflammation during MS is characterized by the accumulation of highly reactive molecules into the extracellular space, such as ROS, RNS, and toxic glutamate, as a consequence of both increased production by activated leukocytes, macrophage, and microglia, and impaired detoxification which is mainly operated by astrocytes [39]. Astrocytes are indeed strongly equipped with antioxidant machineries through which they counteract the failure of cerebral homeostasis in the injured CNS. Additionally, they are actively implicated in the control of the extracellular levels of glutamate, the main excitatory neurotransmitter, which under physiological conditions, is rapidly removed from the microenvironment via specific transporters and converted into glutamine by astrocytes [1]. In MS patients, enhanced concentration of extracellular glutamate, oxidative stress, iron accumulation, mitochondrial injury, and ion channel dysfunction have been documented and indicated among others as causes of neurodegeneration [28]. Together with our previous report showing reduced astrogliosis and demyelination in the EAE Rai^−/−^ mice [16], these findings identify Rai as a driver of IL-17 dependent, disease-promoting astrocyte pathogenic activities.

EVs have been recognized as key mediators of cell–cell communication between neurons, astrocytes, microglia, and oligodendrocytes both under physiological and pathological conditions, and accumulating evidence indicates that ADEVs can regulate neuronal survival and maturation [40]. Interestingly, ADEVs containing apolipoprotein D have been recently shown to be internalized by neurons and to protect them against oxidative stress [41]. Additionally, oxidative stress-induced ADEVs promote the survival of neurons by transferring Synapsin I [42]. At present, only few reports have investigated how specific stimuli impact on the protein composition of ADEVs. Datta Chaudhuri et al. [43] identified a stimulus-dependent protein composition of ADEVs implicated in the support of neurite outgrowth and neuronal survival following ATP or IL-10 stimulation and, conversely, an enrichment in proteins supporting immune cell infiltration following treatment with the pro-inflammatory cytokine IL-1β. Although it has been demonstrated that EVs contribute to neuroinflammation and neurotoxicity in MS [44], to our knowledge, no data are available on the protein composition of ADEVs shed in response to T cell soluble factors [40]. Astrocyte reaction contributes to the remyelination process in MS through the secretion of soluble factors that both support and inhibit oligodendrocyte progenitor cell proliferation, differentiation, and migration [31]. We can hypothesize that Rai, by modulating the protein contents of ADEVs, might modulate neuron and oligodendrocyte function during MS. Indeed, Rai^−/−^ ADEVs contain seven proteins which were enriched when compared with WT ADEVs, of which one is a protein unique to Rai^−/−^ ADEVs, Erp44. Erp44 is a chaperone protein that promotes normal protein folding and among other things is involved in adiponectin folding [45]. Adiponectin, an adipose tissue-derived cytokine, has been found to limit CNS autoimmune inflammation in EAE mice by restraining Th17 differentiation [46] and to limit the LPS-induced pro-inflammatory phenotype of microglia [47]. IL-17-dependent secretion of Erp44 in Rai^−/−^ ADEVs therefore appears as an additional mechanism by which astrocytes lacking Rai limit neuroinflammation by counteracting the pathogenic function of both Th17 cells and microglia. Similar to Erp44, increased levels of microtubule-associated protein 4 (MAP4) and PRDX6 were found in Rai^−/−^ ADEVs compared with control ADEVs in response to IL-17 treatment of astrocytes. MAP4, together with other MAPs, is involved in the regulation of the microtubule network during the differentiation of myelin-forming oligodendrocytes [48]. PRDX6 protects the myelin sheet against oxidative stress [49]. Hence, the higher levels of MAP4 and PRDX6 found in Rai^−/−^ ADEVs further support the notion that astrocytes lacking Rai are able to prevent demyelination during EAE also through the secretion of EVs. These data show for the first time that EVs released by Rai^−/−^ astrocytes in response to an inflammatory stimulus, IL-17, may provide protection against oxidative cellular stress to bystander cells, thereby contributing to prevent demyelination.

Collectively, data obtained in this and our previous studies in primary mouse astrocytes established Rai as a novel participant in the as yet largely unknown signaling pathways driving the astrocyte reaction in response to pro-inflammatory signals, whether Rai subserves other functions in astrocytes is unknown. At present, there are no models with targeted deletion of Rai in astrocytes which will allow to validate Rai as a critical regulator of astrocytes function in vivo.

With the caveat that the role of astrocytic Rai in the healthy brain is unknown, we know that Rai expression in neuron is developmentally regulated and reaches its peak in postmitotic neurons where it promotes survival [50]. Nevertheless, upregulation of Rai expression in neuroblastomas and in brain tumors has been associated with disease progression and poor prognosis indicating that the beneficial role played by this protein in healthy neurons become deleterious under pathological conditions [50]. Molecular adaptors are protein lacking enzymatic activity, which play a key role in coupling surface receptor to intracellular signaling pathways and in the integration of signaling pathways originating from different receptors. As such, they finely regulate the cellular response to environmental clues. To perform their roles, adaptors form low affinity interactions with partner proteins in a flexible and transient manner, thereby ensuring a proper and dynamic control of the intracellular signaling cascades which ultimately lead to the activation of transcription factors. A molecular adaptor may not only amplify a stimulus but also attenuate it depending on the context, the adaptor concentration, and the post-translational modifications, which can dynamically change its function.

In this context, we can speculate that modulation of Rai expression and/or post-translational modifications in astrocytes under different conditions may account, at least in part, for the ability of these cells to control multiple processes in health and disease. It is worth noting that different stress stimuli regulate Rai protein expression in brain tissues and in neuronal cells, suggesting an association between cellular response to a specific insult and modulation of Rai expression [51]. From an evolutionary point of view, the ability of cells to adapt to external stimuli orchestrating, via adaptor proteins, the strength and the duration of signaling cascades is an advantage.

Relevant to MS and EAE, we can speculate that upregulation of Rai in astrocytes during disease course may be a driver of disease. Whether and how Rai expression is modulated in astrocytes under different stimulation/disease stage is an important issue to be addressed.

## 4. Materials and Methods

### 4.1. Mice

C57BL/6J Rai^−/−^ mice generated as described [14,52] and C57BL/6J controls were used. Mice were housed in the animal facility at the University of Siena in pathogen-free and climate-controlled (20 ± 2 °C, relative humidity 55 ± 10%) conditions. The cages were provided with mouse houses and nesting material as environmental enrichment, and mice were provided with water and a pelleted diet ad libitum. Procedures and experimentation were carried out in accordance with the 2010/63/EU Directive and approved by the Italian Ministry of Health.

### 4.2. Primary Astrocyte Culture and Treatments

Astrocytes and microglia were purified from newborn (2-day-old) Rai^−/−^ and C57BL/6J mice as described [53,54] by using the Neural Tissue Dissociation kit (T) (Miltenyi Biotec, Bergisch Gladbach, Germany). Glial cells were cultured in flasks and maintained in Dulbecco’s Modified Eagles Medium (DMEM) supplemented with 10% BCS and 20 U/mL penicillin. After two weeks, the microglia-containing supernatant were collected and plated, while adherent astrocytes were trypsinized and replated. Purity of astrocytes and microglia was assessed by flow cytometry using anti-GFAP mAb (clone GA5, eBioscence) and anti-CD11b mAb (clone M1/70, BD-Biosciences) respectively (% GFAP^+^ > 95%, %CD11b^+^ > 95%).

Treatments of astrocytes were performed in serum free medium with IL-17 (50 ng/mL), a combination of IL-17 (50 ng/mL) and IFNγ (10 ng/mL), or a mixture of IL-1a (3 ng/mL), TNF (30 ng/mL), and C1q (400 ng/mL). Alternatively, the culture medium was replaced with conditioned media from LPS-stimulated microglia or IL-2-stimulated MOG-T cells as described [6,17].

### 4.3. RNA Purification, Reverse Transcription, and qRT-PCR

RNA was purified from astrocytes by using the RNeasy Plus Mini Kit (Qiagen, Venlo, The Netherlands) according to the manufacturer’s instructions, and RNA purity and concentration were measured using QIAxpert (Qiagen, Venlo, Netherlands). Single-strand cDNAs were generated using the iScriptTM cDNA Synthesis Kit (Bio- Rad, Hercules, CA, USA), and qRT-PCR was performed using the SsoFastTM EvaGreen^®^ supermix kit (BIO-RAD, Hercules, CA, USA) and specific pairs of primers for Emp1 (Fwd 5′-3′ GAGACACTGGCCAGAAAAGC; Rev 5′-3′ TAAAAGGCAAGGGAATGCAC), S100a10 (Fwd 5′-3′ CCTCTGGCTGTGGACAAAAT; Rev 5′-3′ CTGCTCACAAGAAGCAGTGG), C3 (Fwd 5′-3′ AGCTTCAGGGTCCCAGCTAC; Rev 5′-3′ GCTGGAATCTTGATGGAGACGC), and Hif-1α (Fwd 5′-3′ TGCTTACACACAGAAATGGCCC; Rev 5′-3′ TATGGCCCGTGCAGTGAAGC). Samples were run in duplicate on 96-well optical PCR plates (Sarstedt AG, Nümbrecht, Germany). Values are expressed as ΔΔCT relative to housekeeping gene GAPDH (Fwd 5′-3′ AACGACCCCTTCATTGAC; Rev 5′-3′ TCCACGACATACTCAGCAC) expression.

### 4.4. Cell Lysis and Immunoblots

Astrocytes were lysed in 1% (*v*/*v*) Triton X-100 in 20 mM Tris-HCl (pH 8), 150 mM NaCl in the presence of Protease Inhibitor Cocktail Set III (Cal BioChem, San Diego, CA, USA), and 0.2 mg Na orthovanadate/mL. Proteins were resolved by SDS-PAGE and transferred to nitrocellulose membrane (GE Healthcare Life Sciences, Marlborough, MA, USA) for immunoblotting analysis. Immunoblots were carried out using the following primary antibodies: anti-HSP70 (clone EP1531Y, Abcam, Cambridge, United Kingdom, cod: ab51052, dilution 1:1000), anti-ENOA (clone 3C8, Abnova, Taipei, Taiwan, cod: H00002023-M03, dilution 1:1000), anti-PhosphoNF-κBp65 (clone 93H1, Cell Signaling Technology, Danvers, MA, USA, cod: 3033, dilution 1:1000), anti-Actin (clone C4, Millipore, Burlington, MA, USA, cod: MAB1501, dilution 1:10,000), anti-NF-κBp65 (clone D14E12, Cell Signaling Technology, Danvers, MA, USA, cod: 8242, dilution 1:1000) and peroxidase-labeled secondary antibodies (anti-rabbit cod: 111-035-144, 1:20,000; and anti-mouse cod:115-035-146, 1:10,000; Jackson ImmunoResearch Laboratories, West Grove, PA, USA). Labeled Abs were detected using the ECL kit (SuperSignal^®^ West Pico Chemiluminescent Substrate, Thermo Scientific, Waltham, MA, USA) and scanned immunoblots were quantified using the ImageJ software.

### 4.5. Viability Assay

Astrocytes were seeded in 6 well-plate (0.5 × 10^6^ astrocytes/well) and treated with 1 mM H_2_O_2_ in serum free medium for 24 h. After, treatment cells were washed two times with PBS and harvested by using trypsin/EDTA solution (Sigma-Merck, Darmstadt, Germany). Cell pellets were resuspended in fresh PBS. The disruption of membrane integrity was determined by adding Propidium Iodide (50 mg/mL) for 1 min. Samples were acquired on Guava Easy Cyte cytometer (Millipore, Burlington, MA, USA) and the percentage of viable cells (PI-negative) was measured and analyzed with FlowJo software (TreeStar Inc., Ashland, OR, USA).

### 4.6. Sample Preparation for Proteomic Analysis

A total of 40 × 10^6^ astrocytes/sample were seeded in 175 cm^2^ flasks (7 × 10^6^ cells/flask) and treated or not with IL-17 (50 ng/mL) in a serum-free medium for 24 h. After the treatments, cells and conditioned media were collected. Astrocyte derived extracellular vesicles (ADEVs) were purified from the cell media by differential ultracentrifugation as described [55]. Briefly, free cells and cellular debris were removed by two centrifugation steps, the first at 300× *g* for 10 min and the second at 100,000× *g* for 30 min at 4 °C. The resulting supernatants were centrifuged at 100,000× *g* for 1.30 h at 4 °C to pellet ADEVs. The pellets were resuspended in PBS and washed by an additional centrifugation at 100,000× *g* for 1.30 h at 4 °C. Then, the pellets were solubilized in a denaturation solution composed of 8 M urea, 2 M thiourea, 4% *w*/*v* 3-[(3-cholamidopropyl) dimethylammonia]-1-propanesulfonate hydrate (CHAPS) and 1% *w*/*v* dithioerythritol (DTE). To prepare cells for proteomic analysis, adherent cells were washed two times with PBS. Cells were collected and washed once in PBS. The resulting pellets were solubilized in a denaturation solution composed of 8 M urea, 2 M thiourea, 4% *w*/*v* 3-[(3-cholamidopropyl) dimethylammonia]-1-propanesulfonate hydrate (CHAPS), and 1% *w*/*v* dithioerythritol (DTE).

Eventually, denaturation solution and traces of bromophenol blue were added to samples carrying the same protein amount in 350 μL solution for the analytical run and 700 μg on average in 450 μL solution for the preparative run.

### 4.7. 2D-Electrophoresis

2DElectrophoresis (2DE) was performed using the Immobiline polyacrylamide system [56]. Immobilized nonlinear pH 3–10 gradient on strips 18 cm in length (GE Healthcare, Uppsala, Sweden) were employed in the first dimensional run. Runs were carried out utilizing the EttanTM IPGphorTM Manifold (GE Healthcare, Uppsala, Sweden) at 16 °C with the following electrical conditions: 30 V for 8 h, 200 V for 2 h, from 200 V to 3500 V in 2 h, 3500 V for 2 h, from 3500 V to 5000 V in 2 h, 5000 V for 3 h, from 5000 V to 8000 V in 1 h, 8000 V for 3 h, from 8000 V to 10,000 V in 1 h, and 10,000 V for the rest of the run until to reach a total of 90,000 VhT. Carrier ampholytes were added to EVs samples at 0.2% for the analytical runs and at 2% for the preparative ones. MS-preparative strips were pre-rehydrated with 350 µL of samples at 16 °C for 12 h at 30 V and successively, the remaining 100 µL were loaded by cup at the cathodic ends, at 16 °C applying the following voltage conditions: 200 V for 8 h, from 200 V to 3500 V in 2 h, 3500 V for 2 h, from 3500 V to 5000 V in 2 h, 5000 V for 3 h, from 5000 V to 8000 V in 1 h, 8000 V for 3 h, from 8000 V to 10,000 V in 1 h, and 10,000 V for 10 h for a total of 90,000 VhT. At the end of the first dimensional run, strips were washed with deionized water and equilibrated with two buffers: the first composed of 6 M urea, 2% *w*/*v* sodium dodecyl sulphate (SDS), 2% *w*/*v* DTE, 30% *v*/*v* glycerol, and 0.05 M Tris-HCl pH 6.8 for 12 min; the second one composed of 6 M urea, 2% *w*/*v* SDS, 2.5% *w*/*v* iodoacetamide, 30% *v*/*v* glycerol, 0.05 M Tris-HCl pH 6.8, and a trace of bromophenol blue for 5 min. The second dimension was then performed at 40 mA/gel constant current on 9–16% SDS polyacrylamide linear gradient gels (size: 18 × 20 cm × 1.5 mm) at 9 °C [56]. Analytical gels were stained with ammoniacal silver nitrate, while preparative gels underwent a mass spectrometry-compatible silver staining [57]; then, both were digitized with Image Scanner III laser densitometer supplied with the LabScan 6.0 software (GE Healthcare, Uppsala, Sweden). Two-dimensional image analysis was performed using Image Master 2D Platinum 6.0 software (GE Healthcare, Uppsala, Sweden). First, an intra class analysis was performed by matching all gels of the same condition to its “Master gel” (4 gels for WTNS, 3 for KONS, 4 for WTIL17, and 3 for KOIL17) chosen by the user taking into consideration the resolution and the number of spots as criteria; secondly, an inter class analysis was performed by matching all “Master gels” to each other. Gel comparison resulted in quantitative and qualitative protein differences, validated by a statistical analysis.

### 4.8. Heatmap and PCA Analysis

In order to visualize the behavior of the differentially abundant spots in the considered conditions, a heatmap analysis was performed using the %Volume values of the statistically significative abundant spots. In particular, the clustering of protein spots was performed using Ward’s clustering method and Euclidean distance. The above-mentioned analysis and the related figures were obtained by RStudio Desktop 1.1.463 (Integrated Development for RStudio, Inc., Boston, MA, USA, https://www.rstudio.com; accessed on 15 December 2020).

Differential spots were also used to perform multivariate analysis by principal component analysis (PCA) simplifying the amount of data (%V variables) by linear transformation. By PCA, it is possible visualize experimental groups in a two-dimensional plane on the basis of the differential spot patterns. PCA was performed by RStudio Desktop 1.1.463 (Integrated Development for RStudio, Inc., Boston, MA, USA, https://www.rstudio.com; accessed on 15 December 2020).

### 4.9. MALDI-TOF-TOF MS—Protein Identification

MS-preparative gels were manually cut to excise electrophoretic spots, which were destained first in a solution of 30 mM potassium ferricyanide and 100 mM sodium sulphate anhydrous, later in 200 mM ammonium bicarbonate and dehydrated in 100% acetonitrile (ACN). The protein spots were then rehydrated and digested overnight at 37 °C in trypsin solution. Digested protein solution was placed on MALDI target, dried, covered with matrix solution of 5 mg/mL α-cyano-4-hydroxycinnamic acid (CHCA) in 50% *v*/*v* ACN and 5% *v*/*v* trifluoroacetic acid (TFA) and dried again. MS analysis was then performed with UltrafleXtreme™ MALDI-ToF/ToF instrument equipped with a 200 Hz smartbeam™ I laser in the positive reflector mode according to defined parameters: 80 ns of delay; ion source 1: 25 kV; ion source 2: 21.75 kV; lens voltage: 9.50 kV; reflector voltage: 26.30 kV; and reflector 2 voltage: 14.00 kV. The applied laser wavelength and frequency were 353 nm and 100 Hz, respectively, and the percentage was set to 46%. Final mass spectra were produced by averaging 1500 laser shots targeting five different positions within the spot. Spectra were acquired automatically and the Flex Analysis software version 3.0 (Bruker) was used for their analysis and for assigning the peaks. The applied software generated a list of peaks up to 200, using a signal-to-noise ratio of 3 as threshold for peak acceptance. Recorded spectra were calibrated using peptides arising from trypsin autoproteolysis as internal standard. The resulting mass lists were filtered for contaminant removal: mass matrix-related ions, trypsin autolysis, and keratin peaks. PMF search was performed using MASCOT (Matrix Science Ltd., London, UK, http://www.matrixscience.com; accessed on 1 May 2021) setting up the following search parameters: Mus musculus as taxonomy, Swiss-Prot/TrEMBL as databases, 100 ppm as mass tolerance, one admissible missed cleavage site, carbamidomethylation (iodoacetamide alkylation) of cysteine as fixed modification, and oxidation of methionine as a variable modification.

### 4.10. Network and Pathway Analysis

Network and pathway analysis were performed submitting the accession number of the identified proteins to the MetaCore 6.8 network building tool (Clarivate Analytics, Philadelphia, PA, USA). This software shows a network of protein interactions, graphically represented by “nodes” (proteins) and “arches” (interactions), by the “shortest-path” algorithm. This algorithm builds a hypothetical network connecting two experimental proteins directly or indirectly using one MetaCore database protein, based on information from scientific literature data and annotated databases of protein interactions and metabolic reactions. The relevant pathway maps were then prioritized according to their statistical significance (*p* ≤ 0.001).

### 4.11. Statistical Analysis

For proteomic data, parametric Student’s *t* test was used to compare the percentage of relative volume (%V) of the 2DE protein spots among the groups. Particularly, only differentially abundant spots with a *p*-value ≤ 0.05 and at least two-fold change in the ratio of the %V means were considered statistically significant. Two-way ANOVA with post hoc Bonferroni’s test was used for all the other experiments in which multiple groups were compared. An unpaired *t*-test was used to determine the statistical significance of differences between two groups. GraphPad Prism Software (Version 8.4.2) was used for statistical analyses. A *p* < 0.05 was considered as statistically significant.

## Data Availability

The data generated in this study are available on request from the corresponding authors.

## Supplementary Materials

The following are available online at https://www.mdpi.com/article/10.3390/ijms22157933/s1.
