# Supplementary material for: Differential Proteomic Analysis of Astrocytes and Astrocytes-Derived Extracellular Vesicles from Control and Rai Knockout Mice: Insights into the Mechanisms of Neuroprotection
Source: Int J Mol Sci. 2021 Jul 25;22(15):7933. doi: 10.3390/ijms22157933 (PMC8348125; doi:10.3390/ijms22157933)
Supplement: Supplementary file 1 [file ijms-22-07933-s001.zip › ijms-1255673-SI.pdf]

International Journal of
Molecular Sciences

MDPI

Supplementary Materials

Differential proteomic analysis of astrocytes and astrocytes-derived extracellular vesicles from control and Rai knockout mice: insights into the mechanisms of neuroprotection

Tommaso Montecchi, Enxhi Shaba, Domiziana De Tommaso, Fabrizio Di Giuseppe, Stefania Angelucci, Luca Bini, Claudia Landi, Cosima Tatiana Baldari and Cristina Ulivieri

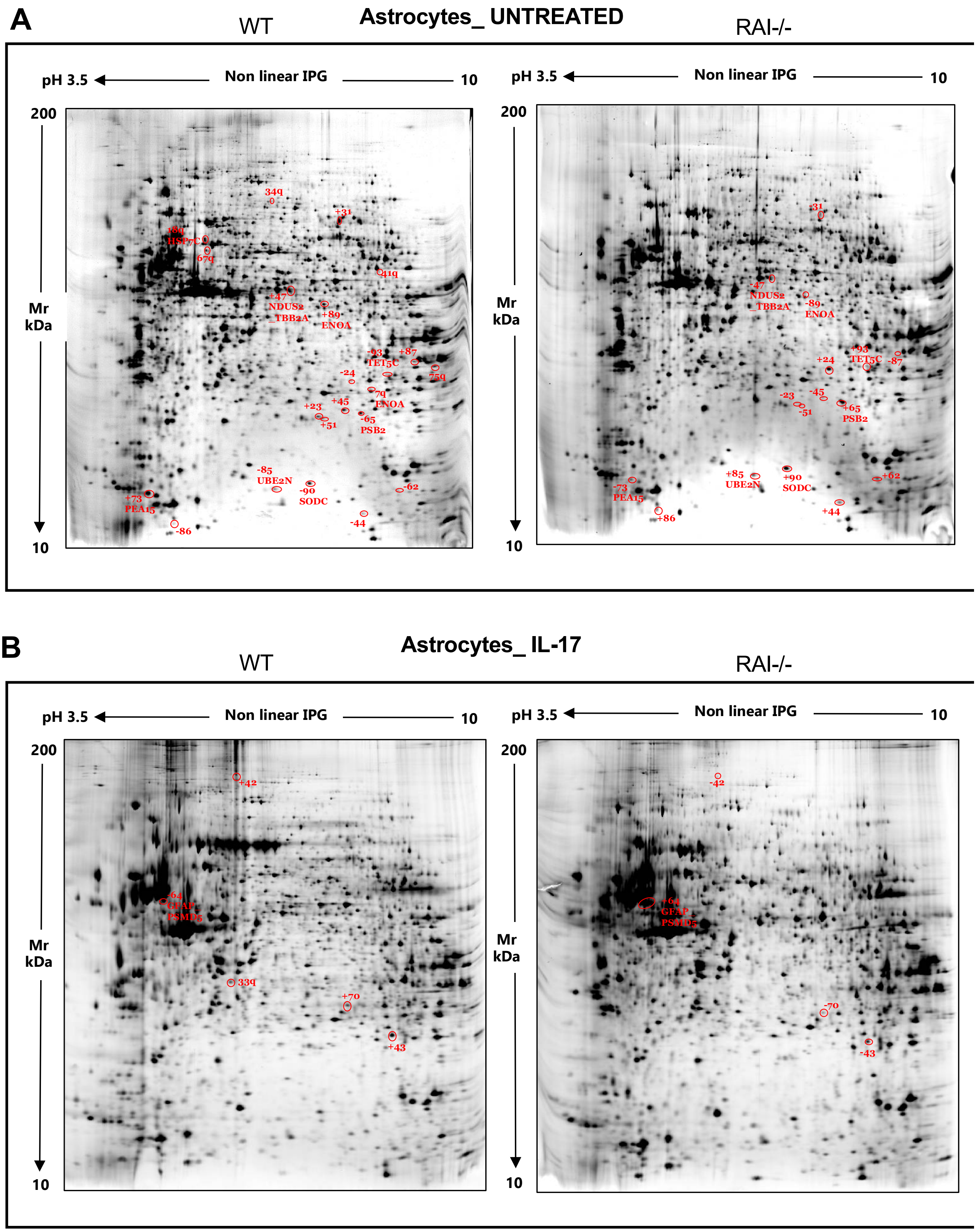

Figure S1. Silver stained electropherograms of astrocytes. Two-dimensional electrophoretic maps of control (WT) and Rai$^{-/-}$ astrocytes untreated (**A**) or treated with IL-17 (50 ng/mL) for 24 h (**B**). Differentially abundant spots by statistical analysis are indicated by numbers and circles.

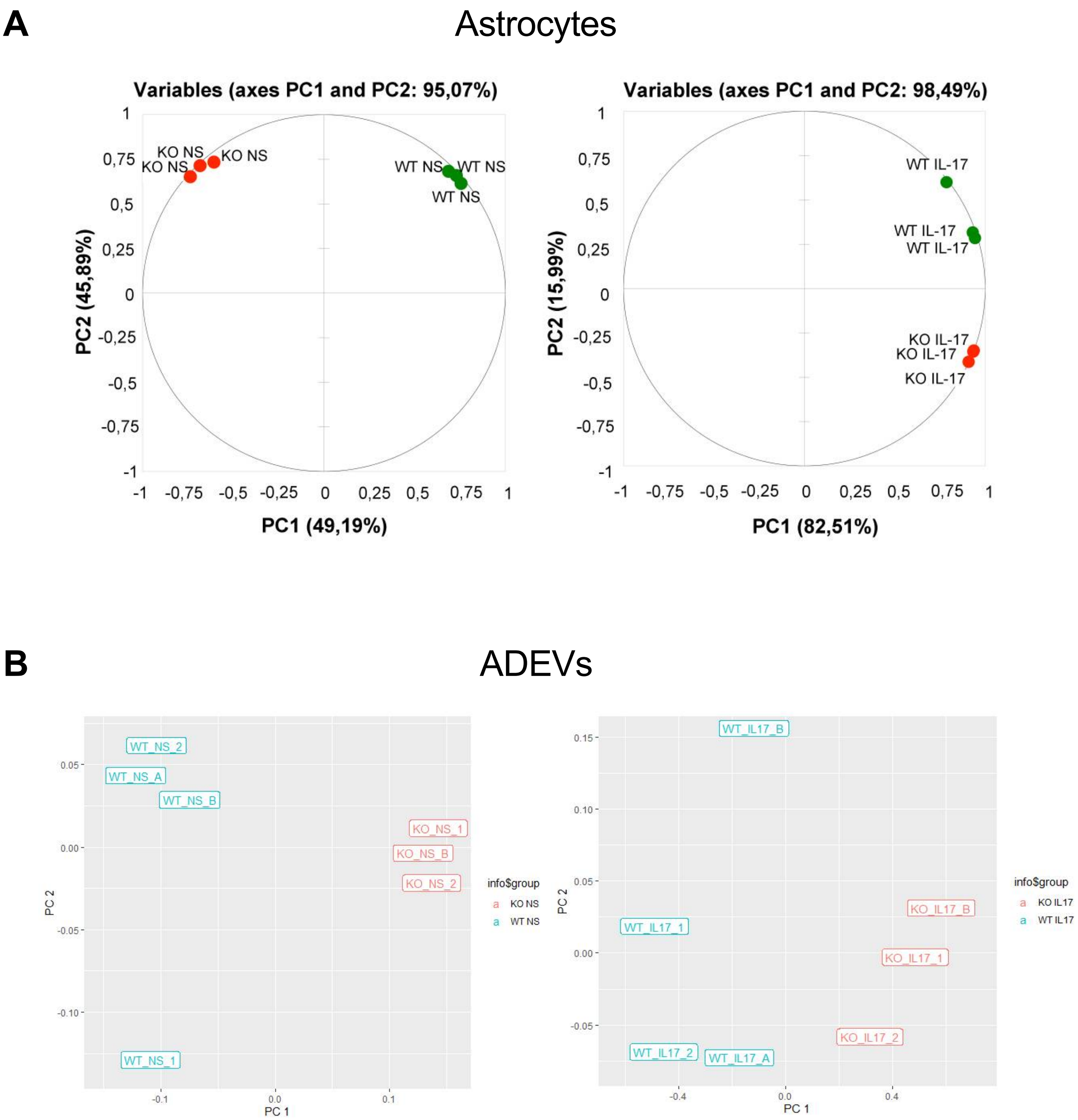

Figure S2. Principal component analysis of astrocytes and ADEVs not treated and IL-17-treated**.** Principal component analysis of differentially abundant spots of WT vs KO not treated (NS) and IL-17-stimulated astrocytes (**A**) and ADEVs (**B**). Figures show that the WT and KO differential spots well correlated to each other within the same WT or KO profile and result well separated between each other, both in the not treated and treated conditions.

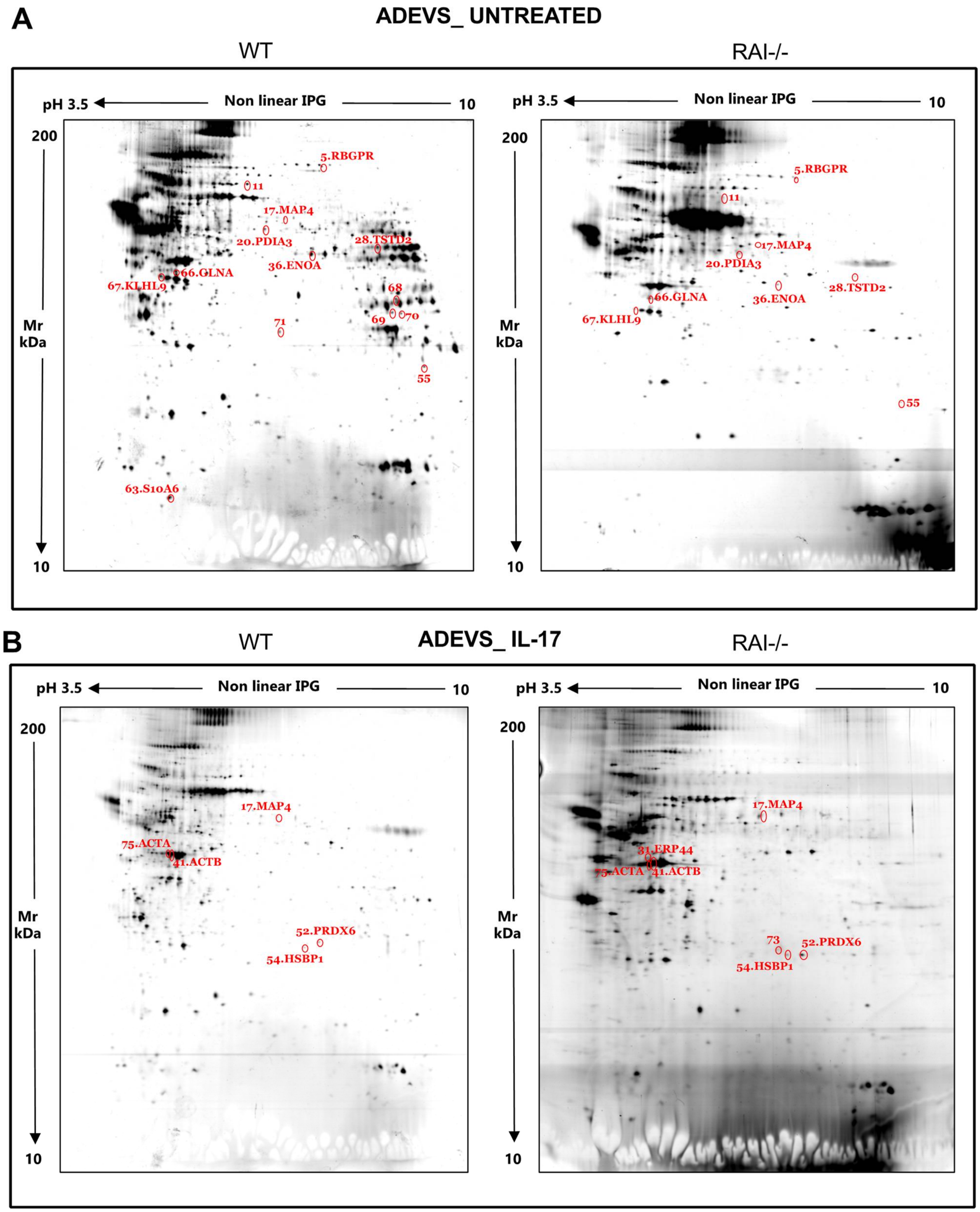

Figure S3. Silver stained electropherograms of ADEVs. Two-dimensional electrophoretic maps of ADEVs isolated from culture media of control (WT) and Rai$^{-/-}$ astrocytes u treated (**A**) or treated with IL-17 (50 ng/mL) for 24 h (**B**). Differentially abundant spots by statistical analysis conditions are indicated by numbers and circles.

Table S1. Differential abundant proteins of astrocytes and ADEVs. Differential abundant proteins (DAPs) of (**A**) astrocytes and (**B**) ADEVs in not stimulated (NS) and IL-17-stimulated Rai wild-type (WT) vs Rai$^{-/-}$ (KO) comparisons. Both table show: number of spot, protein name, abbreviation in UniProt KB, accession number in UniProt KB, theoretical isoelectric point (pI) and molecular weight (MW), MASCOT search results of the identification by MALDI-ToF (score, number of matched peptides out of total peptides, sequence coverage), Kruskal Wallis p-value and fold change for the NS and IL-17 comparison.

A.

Spot Number	Protein name	UniProt Abbreviation	Acession Number	Theoretical pI - MW (kDa)	MASCOT search results			NS			IL-17		
					Score	No. matched peptides	Seq. Coverage	Kruskall Wallis	Rai WT - Rai KO	Rai KO - Rai WT	Kruskall Wallis	Rai WT - Rai KO	Rai KO - Rai WT
7	Alpha-enolase	ENOA	P17182	6.37 - 47453	121	9/12	24%	**3.71E-03**	**0.01 (% V mean)**	0	6.25E-01	0	0
18	Heat shock cognate 71 kDa protein	HSP7C	P63017	5.37 - 71055	195	19/27	36%	**1.42E-02**	**0.05 (% V mean)**	0	5.36E-01	0	0
47	NADH dehydrogenase [ubiquinone] iron-sulfur protein 2, mitochondrial	NDUS2	Q91WD5	6.52 - 52991	199	23/36	48%	**4.29E-02**	**2.60**	0.38	3.76E-01	0.90	1.11
	Tubulin beta-2A chain	TBB2A	Q7TMM9	4.78 - 50274	102	15/36	28%						
64	Glial fibrillary acidic protein	GFAP	P03995	5.27 - 49927	197	22/45	50%	7.02E-02	0.71	1.40	**2.14E-02**	0.43	**2.32**
	26S proteasome non-ATPase regulatory subunit 5	PSMD5	Q8BJY1	5.13 - 56336	175	19/45	45%						
65	Proteasome subunit beta type-2	PSB2	Q9R1P3	6.52 - 23063	236	16/20	53%	**5.75E-03**	0.38	**2.64**	2.07E-01	0.66	1.53
73	Astrocytic phosphoprotein PEA-15	PEA15	Q62048	4.94 - 15102	126	7/7	53%	**4.44E-02**	**3.25**	0.31	1.16E-01	2.50	0.40
85	Ubiquitin-conjugating enzyme E2 N	UBE2N	P61089	6.13 - 17184	153	10/15	56%	**3.50E-02**	0.41	**2.41**	1.35E-01	0.73	1.37
89	Alpha-enolase	ENOA	P17182	6.37 47453	74	27/45	55%	**3.50E-02**	**3.72**	0.27	4.09E-01	1.19	0.84
90	Superoxide dismutase [Cu-Zn]	SODC	P08228	6.02 - 16104	89	5/8	34%	**4.36E-02**	0.44	**2.26**	2.45E-01	0.87	1.15
93	Terminal nucleotidyltransferase 5C	TET5C	Q5SSF7	5.24 - 45225	184	13/18	30%	**2.14E-02**	0.28	**3.55**	2.67E-01	1.22	0.82

B.

Spot Number	Protein name	UniProt Abbreviation	Acession Number	Theoretical pI - MW (kDa)	MASCOT search results			NS			IL-17		
					Score	No. matched peptides	Seq. Coverage	Kruskal Wallis	Rai WT - Rai KO	Rai KO - Rai WT	Kruskal Wallis	Rai WT - Rai KO	Rai KO - Rai WT
5	Rab3 GTPase-activating protein non-catalytic subunit	RBGPR	Q8BMG7	5.84 - 154035	86	11/18	8%	**0.02**	**2.73**	0.37	0.29	3.57	0.28
17	Microtubule-associated protein 4	MAP4	P27546	4.90 - 117927	78	6/6	7%	**0.04**	**6.99**	0.14	**0.02**	0.18	**5.54**
20	Protein disulfide-isomerase A3	PDIA3	P27773	5.88 - 57099	268	23/31	38%	**0.04**	**3.4**	0.29	0.61	1.64	0.61
28	Thiosulfate sulfurtransferase/rhoda nese -like domain-containing protein 2	TSTD2	Q3U269	7.10 - 56833	78	7/9	11%	**0.01**	**34.79**	0.03	0	0	0
31	Endoplasmic reticulum resident protein 44	ERP44	Q9D1Q6	5.09 - 47222	143	11/18	30%	0.12	2.01	0.5	**0.00005**	0	**0.06 (%V mean)**
36	Alpha-enolase	ENOA	P17182	6.37 - 47453	215	14/16	36%	**0.03**	**13.62**	0.07	0.2	0	0.1 (%V mean)
41	Actin, cytoplasmic 1	ACTB	P60710	5.29 - 42052	122	9/14	26%	0.56	0.66	1.52	**0.002**	0.44	**2.3**
52	Peroxiredoxin-6	PRDX6	O08709	5.71 - 24969	195	10/10	44%	0.36	0.37	2.68	**0.03**	0.5	**2**
54	Heat shock factor-binding protein 1	HSBP1	Q9CQZ1	6.12 - 23057	100	6/9	33%	0.76	1.21	0.83	**0.01**	0.37	**2.73**
63	Protein S100-A6	S10A6	P14069	5.30 - 10101	89	4/4	30%	**0.02**	**0.09 (%V mean)**	0	0.14	0.08 (%V mean)	0
66	Glutamine synthetase	GLNA	P15105	6.64 - 42834	85	7/14	13%	**0.01**	**2**	0.5	0.21	2.46	0.41
67	Kelch-like protein 9	KLHL9	Q6ZPT1	6.00 - 70210	84	7/10	8%	**0.002**	**4.36**	0.23	0.79	1.22	0.83
75	Actin, aortic smooth muscle	ACTA	P62737	5.23 - 42381	214	14/17	37%	0.147845	0	0.44 (%V mean)	**0.03**	0.42	**2.38**

Table S2. Pathway maps report of astrocytes. Pathway maps reported by MetaCore, based on all differentially abundant proteins in control (WT) versus Rai$^{-/-}$ (KO) astrocytes in untreated (NS) or IL-17-treated conditions (IL-17).

Enrichment analysis report

Enrichment by Pathway Maps

					WT NS vs KO NS - update 04.21(1)_genelist			
#	Maps	Total	pValue	Min FDR	p-value	FDR	In Data	Network Objects from Active Data
1	Mechanisms of deltaF508 CFTR activation by S-nitrosoglutathione	47	4,347E-06	3,738E-04	4,347E-06	3,738E-04	3	HSC70, SOD1, HSP70
2	Proteolysis Putative ubiquitin pathway	23	1,199E-04	3,055E-03	1,199E-04	3,055E-03	2	E2N(UBC13), HSP70
3	Proteolysis Role of Parkin in the Ubiquitin-Proteasomal Pathway	24	1,308E-04	3,055E-03	1,308E-04	3,055E-03	2	Tubulin beta, HSP70
4	HSP70 and HSP40-dependent folding in Huntington's disease	25	1,421E-04	3,055E-03	1,421E-04	3,055E-03	2	HSC70, HSP70
5	Regulation of degradation of deltaF508-CFTR in CF	39	3,491E-04	6,004E-03	3,491E-04	6,004E-03	2	HSC70, HSP70
6	Immune response HSP60 and HSP70/ TLR signaling pathway	54	6,702E-04	8,234E-03	6,702E-04	8,234E-03	2	E2N(UBC13), HSP70
7	Immune response Antigen presentation by MHC class I, classical pathway	54	6,702E-04	8,234E-03	6,702E-04	8,234E-03	2	PSMB2, HSP70
8	Transcription Negative regulation of HIF1A function	69	1,093E-03	1,174E-02	1,093E-03	1,174E-02	2	HSC70, HSP70
9	Development TGF-beta-induction of EMT via ROS	20	1,457E-02	5,798E-02	1,457E-02	5,798E-02	1	SOD1
10	Regulation of degradation of wtCFTR	20	1,457E-02	5,798E-02	1,457E-02	5,798E-02	1	HSC70
11	Regulation of caspase activity in Huntington's disease	20	1,457E-02	5,798E-02	1,457E-02	5,798E-02	1	HSP70
12	Possible regulation of HSF-1/ chaperone pathway in Huntington's disease	21	1,529E-02	5,798E-02	1,529E-02	5,798E-02	1	HSP70
13	HCV-dependent transcription regulation leading to HCC	21	1,529E-02	5,798E-02	1,529E-02	5,798E-02	1	HSP70
14	HCV-dependent regulation of RNA polymerases leading to HCC	21	1,529E-02	5,798E-02	1,529E-02	5,798E-02	1	HSP70
15	Inflammatory response in ischemia-reperfusion injury during myocardial inf	21	1,529E-02	5,798E-02	1,529E-02	5,798E-02	1	HSP70
16	Cell adhesion Gap junctions	22	1,602E-02	5,798E-02	1,602E-02	5,798E-02	1	Tubulin beta
17	CFTR folding and maturation (normal and CF)	24	1,746E-02	5,798E-02	1,746E-02	5,798E-02	1	HSP70
18	Development Glucocorticoid receptor signaling	25	1,818E-02	5,798E-02	1,818E-02	5,798E-02	1	HSP70
19	CREB1-dependent transcription deregulation in Huntington's Disease	26	1,891E-02	5,798E-02	1,891E-02	5,798E-02	1	SOD1
20	Apoptosis and survival IL-17-induced CIKS-dependent NF-kB signaling a	28	2,035E-02	5,798E-02	2,035E-02	5,798E-02	1	E2N(UBC13)
21	Immune response BAFF-induced non-canonical NF-kB signaling	30	2,179E-02	5,798E-02	2,179E-02	5,798E-02	1	E2N(UBC13)
22	IL-1 beta-dependent CFTR expression	31	2,250E-02	5,798E-02	2,250E-02	5,798E-02	1	E2N(UBC13)
23	Cell cycle Role of Nek in cell cycle regulation	32	2,322E-02	5,798E-02	2,322E-02	5,798E-02	1	Tubulin beta
24	The role of KEAP1/NRF2 pathway in skin sensitization	32	2,322E-02	5,798E-02	2,322E-02	5,798E-02	1	HSP70
25	Apoptosis and survival IL-17-induced CIKS-dependent MAPK signaling p	32	2,322E-02	5,798E-02	2,322E-02	5,798E-02	1	E2N(UBC13)
26	The innate immune response to contact allergens	33	2,394E-02	5,798E-02	2,394E-02	5,798E-02	1	HSP70
27	Signal transduction Erk Interactions: Inhibition of Erk	34	2,466E-02	5,798E-02	2,466E-02	5,798E-02	1	PEA15
28	Oxidative stress ROS-mediated activation of MAPK via inhibition of phosp	34	2,466E-02	5,798E-02	2,466E-02	5,798E-02	1	SOD1
29	Mechanisms of CAM-DR in multiple myeloma	35	2,538E-02	5,798E-02	2,538E-02	5,798E-02	1	HSP70
30	Immune response TLR ligands	35	2,538E-02	5,798E-02	2,538E-02	5,798E-02	1	HSP70
31	Immune response Lipoxins and Resolvin E1 inhibitory action on neutroph	35	2,538E-02	5,798E-02	2,538E-02	5,798E-02	1	SOD1
32	Cytoskeleton remodeling Keratin filaments	36	2,609E-02	5,798E-02	2,609E-02	5,798E-02	1	Tubulin beta
33	Immune response HMGB1/TLR signaling pathway	36	2,609E-02	5,798E-02	2,609E-02	5,798E-02	1	E2N(UBC13)
34	Influence of low doses of Arsenite on Glucose stimulated Insulin secretion	36	2,609E-02	5,798E-02	2,609E-02	5,798E-02	1	SOD1
35	IL-6 signaling in colorectal cancer	37	2,681E-02	5,798E-02	2,681E-02	5,798E-02	1	HSP70
36	Impaired macrophage phagocytic function in asthma	38	2,752E-02	5,798E-02	2,752E-02	5,798E-02	1	SOD1
37	Neutrophil chemotaxis in asthma	38	2,752E-02	5,798E-02	2,752E-02	5,798E-02	1	HSP70
38	Apoptosis and survival Ubiquitination and phosphorylation in TNF-alpha-i	39	2,824E-02	5,798E-02	2,824E-02	5,798E-02	1	E2N(UBC13)
39	Autocrine Somatotropin signaling in breast cancer	39	2,824E-02	5,798E-02	2,824E-02	5,798E-02	1	SOD1
40	Impaired inhibitory action of lipoxins and Resolvin E1 on neutrophil functio	43	3,109E-02	5,798E-02	3,109E-02	5,798E-02	1	SOD1
41	Role of Insulin in regulation of eating behavior in obesity	43	3,109E-02	5,798E-02	3,109E-02	5,798E-02	1	SOD1
42	Immune response BAFF-induced canonical NF-kB signaling	43	3,109E-02	5,798E-02	3,109E-02	5,798E-02	1	E2N(UBC13)
43	Role of TLR signaling in skin sensitization	44	3,181E-02	5,798E-02	3,181E-02	5,798E-02	1	HSP70
44	Apoptosis and survival Inhibition of ROS-induced apoptosis by 17beta-es	44	3,181E-02	5,798E-02	3,181E-02	5,798E-02	1	SOD1
45	Inhibition of remyelination in multiple sclerosis: regulation of cytoskeleton p	44	3,181E-02	5,798E-02	3,181E-02	5,798E-02	1	Tubulin beta
46	HIF-1 in gastric cancer	49	3,536E-02	5,798E-02	3,536E-02	5,798E-02	1	ENO1
47	DNA damage ATM/ATR regulation of G2/M checkpoint: cytoplasmic signa	51	3,678E-02	5,798E-02	3,678E-02	5,798E-02	1	PEA15
48	Immune response Lysophosphatidic acid signaling via NF-kB	52	3,749E-02	5,798E-02	3,749E-02	5,798E-02	1	PEA15
49	Neurophysiological process Synaptic vesicle fusion and recycling in nerve	52	3,749E-02	5,798E-02	3,749E-02	5,798E-02	1	HSC70
50	Oxidative stress Role of ASK1 under oxidative stress	54	3,891E-02	5,798E-02	3,891E-02	5,798E-02	1	SOD1

Enrichment analysis report

Enrichment by Pathway Maps

					WT IL17 vs KO IL17 astrocytes			
#	Maps	Total	pValue	Min FDR	p-value	FDR	In Data	Network Objects from Active Data
1	Immune responses in asthma (schema)	8	1,323E-03	1,093E-02	1,323E-03	1,093E-02	1	IL-17
2	Intercellular relations in asthma (general schema)	16	2,645E-03	1,093E-02	2,645E-03	1,093E-02	1	IL-17
3	Development_Schema: Adult neurogenesis in the Subventricular Zone	19	3,141E-03	1,093E-02	3,141E-03	1,093E-02	1	GFAP
4	Development_Dopamine-induced expression of CNTF in adult neurogenesis	22	3,637E-03	1,093E-02	3,637E-03	1,093E-02	1	GFAP
5	Role of IL-17-producing T cells in allergic contact dermatitis	23	3,802E-03	1,093E-02	3,802E-03	1,093E-02	1	IL-17
6	Cytoskeleton remodeling_Neurofilaments	25	4,132E-03	1,093E-02	4,132E-03	1,093E-02	1	GFAP
7	Immune response_T cell subsets: secreted signals	25	4,132E-03	1,093E-02	4,132E-03	1,093E-02	1	IL-17
8	Role of fibroblasts and keratinocytes in the elicitation phase of allergic contact dermatitis	26	4,297E-03	1,093E-02	4,297E-03	1,093E-02	1	IL-17
9	Role of Th17 cells in asthma	28	4,627E-03	1,093E-02	4,627E-03	1,093E-02	1	IL-17
10	Th1 and Th17 cells in an autoimmune mechanism of emphysema formation in smokers	28	4,627E-03	1,093E-02	4,627E-03	1,093E-02	1	IL-17
11	Impaired inhibition of Th17 cell differentiation by IFN-beta in multiple sclerosis	28	4,627E-03	1,093E-02	4,627E-03	1,093E-02	1	IL-17
12	IL-17 and IL-17F-induced inflammatory signaling in normal and asthmatic airway epithelium	28	4,627E-03	1,093E-02	4,627E-03	1,093E-02	1	IL-17
13	Development_Astrocyte differentiation (general schema)	28	4,627E-03	1,093E-02	4,627E-03	1,093E-02	1	GFAP
14	Apoptosis and survival_IL-17-induced CIKS-dependent NF-kB signaling and mRNA stabilization	28	4,627E-03	1,093E-02	4,627E-03	1,093E-02	1	IL-17
15	T regulatory cells in asthma	30	4,957E-03	1,093E-02	4,957E-03	1,093E-02	1	IL-17
16	Putative role of Tregs in COPD	30	4,957E-03	1,093E-02	4,957E-03	1,093E-02	1	IL-17
17	Inter-cellular relations in COPD (general schema)	30	4,957E-03	1,093E-02	4,957E-03	1,093E-02	1	IL-17
18	Immune response_IL-23 signaling pathway	31	5,122E-03	1,093E-02	5,122E-03	1,093E-02	1	IL-17
19	Neutrophil adhesion and transendothelial migration in asthma	31	5,122E-03	1,093E-02	5,122E-03	1,093E-02	1	IL-17
20	Apoptosis and survival_IL-17-induced CIKS-dependent MAPK signaling pathways	32	5,287E-03	1,093E-02	5,287E-03	1,093E-02	1	IL-17
21	Immune response_IL-11 signaling via JAK/STAT	34	5,617E-03	1,093E-02	5,617E-03	1,093E-02	1	GFAP
22	Proinflammatory cytokine release from eosinophils in asthma	34	5,617E-03	1,093E-02	5,617E-03	1,093E-02	1	IL-17
23	Interleukins-induced inflammatory response in asthmatic airway fibroblasts	35	5,782E-03	1,093E-02	5,782E-03	1,093E-02	1	IL-17
24	Immune response_Th17 cell differentiation	35	5,782E-03	1,093E-02	5,782E-03	1,093E-02	1	IL-17
25	Development_Schema: Adult neuron differentiation in the Subventricular and Subgranular Zones	35	5,782E-03	1,093E-02	5,782E-03	1,093E-02	1	GFAP
26	Th17 cytokines in COPD	36	5,947E-03	1,093E-02	5,947E-03	1,093E-02	1	IL-17
27	Eosinophil-derived cytokines in airway remodeling in asthma	38	6,277E-03	1,093E-02	6,277E-03	1,093E-02	1	IL-17
28	TNF-alpha-induced inflammatory signaling in normal and asthmatic airway epithelium	38	6,277E-03	1,093E-02	6,277E-03	1,093E-02	1	IL-17
29	Development_Neural stem cell lineage commitment (schema)	38	6,277E-03	1,093E-02	6,277E-03	1,093E-02	1	GFAP
30	Immune response_Th17, Th22 and Th9 cell differentiation	39	6,442E-03	1,093E-02	6,442E-03	1,093E-02	1	IL-17
31	Development_Astrocyte differentiation from adult stem cells	40	6,607E-03	1,093E-02	6,607E-03	1,093E-02	1	GFAP
32	Stem cells_NOTCH1-induced self-renewal of glioblastoma stem cells	40	6,607E-03	1,093E-02	6,607E-03	1,093E-02	1	GFAP
33	Role of Bregs in attenuation of T and NK cells mediated anti-tumor immune responses	41	6,772E-03	1,093E-02	6,772E-03	1,093E-02	1	IL-17
34	Regulation of proinflammatory cytokine production by Th2 cells in asthma	43	7,102E-03	1,093E-02	7,102E-03	1,093E-02	1	IL-17
35	NF-kB-, AP-1- and MAPKs-mediated proinflammatory cytokine production by eosinophils in asthma	43	7,102E-03	1,093E-02	7,102E-03	1,093E-02	1	IL-17
36	Release of pro-inflammatory factors and proteases by alveolar macrophages in asthma	44	7,266E-03	1,093E-02	7,266E-03	1,093E-02	1	IL-17
37	Dual function of Treg cells in cancer development	46	7,596E-03	1,093E-02	7,596E-03	1,093E-02	1	IL-17
38	Immune response_Naive CD4+ T cell differentiation	46	7,596E-03	1,093E-02	7,596E-03	1,093E-02	1	IL-17
39	Apoptosis and survival_IL-17-induced CIKS-independent signaling pathways	46	7,596E-03	1,093E-02	7,596E-03	1,093E-02	1	IL-17
40	IL-17-induced mucin expression in CF airways	48	7,926E-03	1,093E-02	7,926E-03	1,093E-02	1	IL-17
41	PDE4 regulation of cyto/chemokine expression in arthritis	49	8,090E-03	1,093E-02	8,090E-03	1,093E-02	1	IL-17
42	Neutrophil-derived granule proteins and cytokines in asthma	49	8,090E-03	1,093E-02	8,090E-03	1,093E-02	1	IL-17
43	Breakdown of CD4+ T cell peripheral tolerance in type 1 diabetes mellitus	49	8,090E-03	1,093E-02	8,090E-03	1,093E-02	1	IL-17
44	Th17 cells in CF (mouse model)	49	8,090E-03	1,093E-02	8,090E-03	1,093E-02	1	IL-17
45	PDE4 regulation of cyto/chemokine expression in inflammatory skin diseases	50	8,255E-03	1,093E-02	8,255E-03	1,093E-02	1	IL-17
46	Rheumatoid arthritis (general schema)	50	8,255E-03	1,093E-02	8,255E-03	1,093E-02	1	IL-17
47	Inflammatory factors-induced expression of mucins in normal and asthmatic epithelium	51	8,420E-03	1,093E-02	8,420E-03	1,093E-02	1	IL-17
48	Development_Self-renewal of adult neural stem cells	52	8,585E-03	1,093E-02	8,585E-03	1,093E-02	1	GFAP
49	Immune response_T cell subsets: cell surface markers	52	8,585E-03	1,093E-02	8,585E-03	1,093E-02	1	IL-17
50	Proinflammatory cytokine production by Th17 cells in asthma	53	8,749E-03	1,093E-02	8,749E-03	1,093E-02	1	IL-17

Table S3. Pathway maps report of ADEVs. Pathway maps reported by MetaCore, based on all differentially abundant proteins in ADEVs released from control (WT) versus Rai$^{-/-}$ (KO) astrocytes untreated (NS) or treated with IL-17 (IL-17).

Enrichment analysis report

Enrichment by Pathway Maps — WT NS vs KO NS Evs

#	Maps	Total	pValue	Min FDR	p-value	FDR	In Data	Network Objects from Active Data
1	p53 signaling in Prostate Cancer	33	1,337E-02	3,570E-02	1,337E-02	3,570E-02	1	MAP4
2	Beta-catenin-dependent transcription regulation in colorectal cancer	36	1,458E-02	3,570E-02	1,458E-02	3,570E-02	1	Calcyclin
3	Nitrogen metabolism	36	1,458E-02	3,570E-02	1,458E-02	3,570E-02	1	GLNA
4	WNT signaling in HCC	40	1,619E-02	3,570E-02	1,619E-02	3,570E-02	1	GLNA
5	Development_Role of Thyroid hormone in regulation of oligodendrocyte di	48	1,940E-02	3,570E-02	1,940E-02	3,570E-02	1	GLNA
6	HIF-1 in gastric cancer	49	1,980E-02	3,570E-02	1,980E-02	3,570E-02	1	ENO1
7	Neurophysiological process_GABAergic neurotransmission	51	2,060E-02	3,570E-02	2,060E-02	3,570E-02	1	GLNA
8	Immune response_Antigen presentation by MHC class I, classical pathway	54	2,180E-02	3,570E-02	2,180E-02	3,570E-02	1	PDIA3
9	Role of Thyroid hormone in regulation of oligodendrocyte differentiation in	55	2,220E-02	3,570E-02	2,220E-02	3,570E-02	1	GLNA
10	Development_Regulation of cytoskeleton proteins in oligodendrocyte diffe	59	2,380E-02	3,570E-02	2,380E-02	3,570E-02	1	MAP4
11	Cell adhesion_Plasmin signaling	70	2,819E-02	3,723E-02	2,819E-02	3,723E-02	1	ENO1
12	Signal transduction_mTORC1 upstream signaling	74	2,978E-02	3,723E-02	2,978E-02	3,723E-02	1	PDIA3
13	Glycolysis and gluconeogenesis	94	3,771E-02	3,810E-02	3,771E-02	3,810E-02	1	ENO1
14	Transcription_HIF-1 targets	95	3,810E-02	3,810E-02	3,810E-02	3,810E-02	1	ENO1
15	Histidine-glutamate-glutamine metabolism	95	3,810E-02	3,810E-02	3,810E-02	3,810E-02	1	GLNA

Enrichment analysis report

Enrichment by Pathway Maps — WT IL17 vs KO IL17 Evs

#	Maps	Total	pValue	Min FDR	p-value	FDR	In Data	Network Objects from Active Data
1	Possible regulation of HSF-1/ chaperone pathway in Huntington's disease	21	6,825E-03	2,229E-02	6,825E-03	2,229E-02	1	PLA2
2	p53 signaling in Prostate Cancer	33	1,071E-02	2,229E-02	1,071E-02	2,229E-02	1	MAP4
3	Dysregulation of Adiponectin secretion from adipocytes in obesity, type 2	38	1,233E-02	2,229E-02	1,233E-02	2,229E-02	1	ERp44
4	Retinal ganglion cell damage in glaucoma	45	1,458E-02	2,229E-02	1,458E-02	2,229E-02	1	NSGPeroxidase
5	Glucocorticoids-mediated inhibition of pro-constrictory and pro-inflammator	49	1,587E-02	2,229E-02	1,587E-02	2,229E-02	1	PLA2
6	Development_Regulation of cytoskeleton proteins in oligodendrocyte diffe	59	1,909E-02	2,229E-02	1,909E-02	2,229E-02	1	MAP4
7	Plasmalogen biosynthesis	64	2,069E-02	2,229E-02	2,069E-02	2,229E-02	1	PLA2
8	Oxidative stress in adipocyte dysfunction in type 2 diabetes and metabolic	64	2,069E-02	2,229E-02	2,069E-02	2,229E-02	1	PLA2
9	TNF-alpha and IL-1 beta-mediated regulation of contraction and secretion	65	2,101E-02	2,229E-02	2,101E-02	2,229E-02	1	PLA2
10	Immune response_CD16 signaling in NK cells	69	2,229E-02	2,229E-02	2,229E-02	2,229E-02	1	PLA2

Table S4. Pathway maps report of comparison between astrocytes and ADEVs. Pathway maps reported by MetaCore, based on all differentially abundant proteins in control (WT) astrocytes and ADEVs versus Rai-/- (KO) astrocytes and ADEVs.

Enrichment by Pathway Maps					WT NS vs KO NS Astrocytes				WT NS vs KO NS Evs			
#	Maps	Total	min(pValue)	Min FDR	p-value	FDR	In Data	Network Objects from Active Data	p-value	FDR	In Data	Network Objects from Active Data
1	Immune response_Antigen presentation by MHC class I, classical pathway	54	6.702E-04	8.234E-03	6.702E-04	8.234E-03	2	PSMB2, HSP70	2.180E-02	3.570E-02	1	PDIA3
2	HIF-1 in gastric cancer	49	1.980E-02	3.570E-02	3.536E-02	5.798E-02	1	ENO1	1.980E-02	3.570E-02	1	ENO1
3	Development_Regulation of cytoskeleton proteins in oligodendrocyte differentiation and myelination	59	2.380E-02	3.570E-02	4.244E-02	5.798E-02	1	Tubulin beta	2.380E-02	3.570E-02	1	MAP4
4	Cell adhesion_Plasmin signaling	70	2.819E-02	3.723E-02	5.018E-02	5.831E-02	1	ENO1	2.819E-02	3.723E-02	1	ENO1
5	Glycolysis and gluconeogenesis	94	3.771E-02	3.810E-02	6.686E-02	7.172E-02	1	ENO1	3.771E-02	3.810E-02	1	ENO1
6	Transcription_HIF-1 targets	95	3.810E-02	3.810E-02	6.755E-02	7.172E-02	1	ENO1	3.810E-02	3.810E-02	1	ENO1
